# Executive Functions Assessment in a Child with Autism: A Pilot Single-Case Study from a Longitudinal and Mixed Methods Approach

**DOI:** 10.3390/children11121468

**Published:** 2024-11-30

**Authors:** Marian Acero-Ferrero, Elena Escolano-Pérez

**Affiliations:** Faculty of Education, Department of Psychology and Sociology, University of Zaragoza, 50009 Zaragoza, Spain; macero@unizar.es

**Keywords:** autism spectrum disorder, intervention, assessment, mixed methods, systematic observation, lag sequential analysis

## Abstract

Although the literature confirms executive deficits in individuals with autism spectrum disorder (ASD) that hinder adaptation, evidence-based intervention programs targeting this area are scarce, and even fewer have evaluated their effectiveness. Objectives: This study aimed to assess a pilot program designed to improve the executive functions of a child five years and nine months in age with ASD. Methods: To evaluate the effects of the intervention, observational methodology was used, which is considered a mixed method in itself as it integrates both qualitative and quantitative elements in its various phases. Specifically, an idiographic, longitudinal, and multidimensional design was followed. A lag sequential analysis was conducted using GSEQ software Version 5.1, enabling us to study changes in the executive functions of a child before, during, and after the intervention, including whether its effects are sustained over time. Results: The sequential patterns obtained indicate more appropriate and complex executive functioning after the three-month intervention, specifically increased cognitive flexibility, improved self-regulation, more accurate evaluation, and progress in inhibitory processes. However, these improvements do not persist over time. Conclusion: This study contributes to the scarcely explored field of executive function interventions in children with ASD, although it is necessary to consider the generalization of the results to other contexts, such as family and school, during interaction with peers.

## 1. Introduction

Executive functions (EFs) are a set of high-level cognitive processes that enable goal-directed actions, essential for adaptation in new, complex, or confusing situations [1]. Despite numerous models of EF—some of which also include high-level affective processes [2]—the most widely accepted model that best explains their development posits the existence of three main components [1,3,4,5]: working memory (related to the simultaneous processing and storage of information); flexibility (involving the quick and effective shifting of attention); and inhibition (the suppression of predominant information or overlearned responses that are irrelevant to the goal). Executive functions are present in our daily lives, and they are essential for independent functioning, for example, when we go to the supermarket to buy the ingredients for a recipe without making a list beforehand, and we have just enough money to do so. Working memory is needed to remember all the ingredients we need to buy to cook the recipe; flexibility to be able to look for a similar product in case the one we are looking for is not available; and inhibition to be able to resist temptation while shopping in case we find products on sale that appeal to us which we like or that we fancy at that moment because we are hungry and are not able to buy them because we have just enough money. From these distinct but interrelated executive functions, more complex ones develop, such as reasoning, problem-solving, and planning. Increasingly, studies on EFs in children highlight their importance for health, as well as for school, academic, and social adjustment [1,6]. However, despite this growing interest in children’s EF, much remains to be understood, particularly regarding how EF develops and its potential for significant improvement through targeted training [7].

Another series of studies on EF that are also becoming more numerous focuses on individuals with Autism Spectrum Disorder (ASD) from Western and Eastern countries and in both developed and developing countries [8,9]. This is because these individuals exhibit cognitive difficulties, with executive function challenges being among the most common [10,11,12,13]. These difficulties are evident as early as preschool age [6,12,14]. Several studies indicate that another frequent cognitive deficit in people with ASD is in Theory of Mind—the ability to interpret others’ beliefs, intentions, and emotions. Studies have attempted to determine the link between executive functions and mental abilities both in clinical and non-clinical conditions. This relationship remains unclear. One of the strongest hypotheses is that the Theory of Mind develops very early during childhood and that these skills are necessary to control thoughts, behaviors, and emotions in an interactive and strategic way. This process of maturation of executive skills develops progressively, even extending beyond adolescence. Therefore, it would be the mentalistic skills that would modulate executive control [15,16,17,18,19,20,21,22]. This has contributed to the scarcity of evidence-based intervention programs specifically aimed at improving cognitive abilities in people with ASD, with most lacking scientific grounding, methodological rigor, and adequate evaluation to determine their efficacy [7,17,18,19]. Currently, there is growing evidence, though with certain limitations—among them those related to methodology, heterogeneity characterized in ASD, and generalization and maintenance data—indicating that executive skills training might be effective for children with ASD [23,24,25].

Some studies suggest moving away from distinctions within ASD based on clinical criteria and instead establishing differential profiles of ASD subtypes based on cognitive and/or linguistic functioning levels [26,27]. Supporting this dimensional approach, the DSM-5 suggests a single designation of ASD for diagnosis, accompanied by a description of the functioning profile. Observational methodology allows capturing this functioning, as studying the individual’s habitual and spontaneous behavior in their natural environment enables a detailed, intensive, microgenetic analysis of the process involved [28,29,30,31,32]. This methodology has already been successfully used in previous studies with children with ASD [33,34,35,36,37]. These studies highlight the great advantage of using the mixed-method perspective since it allows us to capture reality as it happens, systematize it, guarantee its quality, and treat it quantitatively in a rigorous manner, regardless of the area of child development under study (for example, social or cognitive skills). This is possible due to the combination of high scientific rigor and flexibility that characterizes the observational methodology [38]. Systematic observation has also been successfully used in the study of children’s EF [39,40,41]. Studying EF through observational methodology also addresses the criticism directed at the use of classical tests for ASD diagnosis and EF evaluation due to their lack of ecological validity, as the artificial situations they create differ significantly from real-world demands and everyday functioning [42]. Therefore, it is necessary to improve and deepen the study of EF in children with ASD, proposing interventions that follow recommendations about evidence-based practices (EBPs) [18,43] and have research evidence of their effectiveness. Thus, it is necessary to rethink issues related to their measurement [44].

Therefore, the aim of this study was to evaluate a cognitive intervention aimed at improving the EF of a child with ASD implemented in a school setting using a mixed-methods approach.

The postulated hypothesis is as follows: The designed cognitive intervention will positively impact the child’s EFs (working memory, flexibility, and inhibition). They will be improved in terms of complexity and elaboration.

## 2. Materials and Methods

### 2.1. Participant

The participant was a boy 5 years and 9 months of age with ASD, previously diagnosed according to DSM-5 criteria [45] and the Autism Diagnostic Observation Schedule-2 (ADOS-2) [46]. The child did not have any comorbidities. He was attending an urban public school with preferential attention to children with ASD. He received specific support from a special education teacher in and out of his classroom. He was an only child, and both his parents were employed. The child was recruited from his own school. After an informational talk about the intervention in executive functions in ASD at the school, the parents agreed to have their child participate in the intervention and signed a written informed consent form.

The initial assessment, conducted by the first author, indicated that the participant presented (1) a total intelligence quotient (IQ) of 119, which implies a superior level of intelligence; a verbal IQ of 104; and a manipulative IQ of 127, according to results from the Wechsler Preschool and Primary Scale of Intelligence—Fourth Edition (WPPSI-IV) [47]; (2) a normal linguistic level, according to results from the Navarra Oral Language Test Revised (PLON-R) [48]; (3) deficiencies in the social dimension (difficulties in understanding social subtleties and low empathy); discourse with limitations in the flexible adaptation of conversations and topics, and extreme perfectionism that caused difficulties in recognizing his mistakes, according to results obtained with the Autism Spectrum Inventory communication and language scale (IDEA, from its Spanish name Inventario de Espectro Autista) [49]; (4) difficulties in certain EFs—specifically in cognitive flexibility (STen score = 4; intermediate–low score) and inhibition (STen score = 2; very low score) but not in working memory (STen score = 5)—according to performance on the Color Trails task, Interference task, and Rings task, respectively, from the ENFEN Battery—Neuropsychological Evaluation of Executive Functions in Children [50].

The participant was treated in accordance with international ethical standards (1964 Helsinki Declaration and its later amendments), as well as European regulations (General Data Protection Regulation, 2016/679) and Spanish data protection laws (Organic Law 3/2018, of December 5, on the Protection of Personal Data and Guarantee of Digital Rights). The research was approved by the Research Ethics Committee of the Autonomous Community of Aragon (identification number PI23/486) and endorsed by the management teams of the schools that the participants attended. The parents of the participant signed a written informed consent form.

### 2.2. Instruments

**Figure 1:**
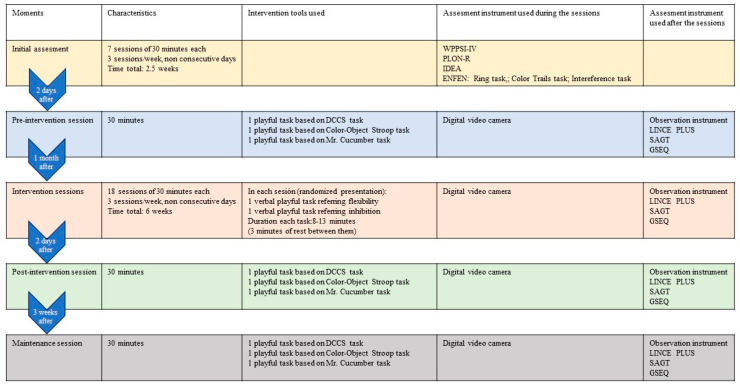
Procedure, Intervention, and assessment tools used in each phase.

The intervention tools used, as well as those for assessments, were the following (see Figure 1).

Intervention tools:

- Intervention instrument: It consisted of a low-intervention program [33,51] to optimize cognitive flexibility and inhibition EF (since these were the affected EF, according to the initial evaluation of the participant). The intervention was based on board games that required the activation of these EFs for their resolution. Given that there are no pure tasks in EF assessment [52], board games have been identified as one of the best ways to work on EF in autism, as playing any type of board game requires activating executive functions internally [53]. The intervention program design lasted for a month and involved three experts in ASD and EF with over 10 years of experience in research and intervention in both areas. Recommendations on evidence-based practices were considered during its development [18,43]. A play-based approach was used as a pivotal approach in the intervention. Play has an important role in early social and cognitive development. Through play, children understand the world, express their knowledge, and interact with others [54]. During the preschool period, children develop their play skills, which is why it has been included in most early intervention programs for children with ASD [53].
- Tasks used in pre- and post-intervention sessions and in the maintenance session: Playful tasks based on (1) the Dimensional Change Card Sort (DCCS) task [55] assessing cognitive flexibility; (2) the Color–Object Stroop task [56] assessing inhibition; and (3) the Mr. Cucumber task [57,58] assessing working memory. Three tasks based on each of them were used, varying the specific elements used as stimuli in each case. In the DCCS standard version, children are required to sort a series of bivalent test cards, first according to one dimension (e.g., color) and then according to the other (e.g., shape). In the border version, children are required to play the color game if there is a border in the card, and if there is no border, children must play the shape game. In the Color-object interference Stroop task, children are shown line drawings of familiar objects in a color that is congruent (e.g., an orange carrot), incongruent (e.g., a green carrot), or neutral (for objects having no canonical color, e.g., a red book]), and abstract shapes, each drawn in one of six colors. The children are asked to name the color in which each object is drawn. In the Mr. Cucumber Test, the outline of an extraterrestrial figure, to which colored stickers had been attached, is displayed. There are three items at each level from 1 to 8 (i.e., with stickers in 1 to 8 positions). The exposure time is 5 seconds, except for items at levels higher than 5; these are exposed far as many seconds as there are stickers attached. The subject must then show, on an outline without colored stickers, the positions of the stickers. The test is discontinued when a subject fails all three items at one level.

Assessment tools:

- Instruments used in the initial assessment: The following instruments were employed to understand the participant’s cognitive profile and to design the intervention program according to their characteristics and needs: (1) Wechsler Preschool and Primary Scale of Intelligence—Fourth Edition (WPPSI-IV), Spanish validation [47]; (2) Revised Navarra Oral Language Test (PLON-R) [48]; and (3) Autism Spectrum Inventory communication and language scale (IDEA) [49]; (4) ENFEN Battery—Neuropsychological Assessment of Executive Functions in Children—[50]. Although this battery consists of four tests aimed at assessing different EF, this study focused only on those related to (1) working memory (Rings task), (2) cognitive flexibility (Color Trails task), and (3) inhibition (Interference task). This choice was consistent with the theoretical proposal of this study, which argues that these three processes are the main components of EF.
- Technical instrument for observational data collection: A digital video camera was used to record the observation sessions (i.e., the pre-intervention session, intervention sessions, post-intervention session, and maintenance session).
- Observation instrument: Given the enormous variety of typologies of behaviors that an individual can perform spontaneously, all of them could not be collected with a sufficient degree of detail and specification in a standardized instrument. Consequently, observational methodology requires construct ad hoc the observation instrument that allows observing with sufficient detail and degree of specification the behaviors that are the object of interest in each study [28,59]. Therefore, in this research, an observation instrument was ad hoc constructed to observe behaviors indicative of EF use, as well as other complementary processes (self-control) and characteristics of child action (execution quality: correct, incorrect, etc.), in addition to adult mediating actions. Given the study’s objective and multidimensional design, the observation instrument built was of field format, specifically mixed (Table 1)—complementarity between field format and category system. For its construction, the following were considered: (1) the object of study reality (recordings of preliminary sessions with another child similar to the participant in this study); (2) existing literature on EF development, especially in children with ASD [15,60,61]; (3) literature on effective intervention principles in these children [18,43,62]; (4) other observation instruments previously constructed by other authors for the study of child cognitive development [29,35,36,40]. A continuous development and revision process was followed until the final version of the observation instrument was reached [59], which consisted of 9 criteria and 19 categories (Table 1).

Instrument for recording observational data: The free software LINCE PLUS [63], https://observesport.github.io/lince-plus/ (accessed on 27 November 2024) was used.

- Instruments for the analysis of observational data: (1) The free software Software Application for Generalizability Theory (SAGT) v1.0 [64] was used to calculate the quality of observational data http://d8.lcc.uma.es/menpas/ElegirCuestionarios.aspx (accessed on 27 November 2024); (2) The free software GSEQ Version 5.1 [65] https://www.mangold-international.com/en/products/software/gseq.html (accessed on 27 November 2024) was used to obtain behavioral patterns.

### 2.3. Procedure

Systematic observation was used to assess child activity during the pre-intervention session, the intervention (at three points of implementation), the post-intervention session, as well as during the maintenance session. This methodology allows for a rigorous and detailed evaluation of child behavior in the natural context where the intervention takes place [65,66,67,68], overcoming criticisms regarding the lack of ecologically valid assessments in interventions with individuals with ASD [42]. The participant’s age justified the use of observational methodology to address the aim of this study. Additionally, this methodology is intensive and involves working with a reduced number of participants [33,69,70,71]. Systematic observation is currently considered a mixed-methods approach itself, as it allows for the integration of qualitative and quantitative elements in the QUAL-QUAN-QUAL sequence of phases. In the first QUAL phase, the study objective is determined, the observational design to be followed is established, and the observation instrument is constructed ad hoc. Its application to the study reality, and therefore, the coding of observed behaviors based on an order or sequence criterion, leads to the QUAN phase. In this phase, the initial qualitative dataset can be transformed into quantitative data using different techniques—such as lag sequential analysis. This allows for obtaining quantitative results as sequential behavior patterns. Subsequently, the last QUAL stage begins with the interpretation of the results, considering the initial study problem (raised in the first QUAL stage), allowing for seamless integration [38,72,73,74,75].

Considering the eight existing observational designs [28], the design used in this study was idiographic (since a single observational unit was studied, specifically, the child with ASD receiving the intervention), longitudinal (21 observation sessions were conducted distributed across four temporal moments), and multidimensional (several dimensions of behavior related to different EF and other relevant aspects of child action and adult mediating actions were observed).

In all sessions, the participant and the same researcher were present. The distribution and characteristics of the sessions were as follows:

- Before designing the intervention program, and therefore, before its implementation, the initial evaluation of the participant was conducted. A total of seven sessions of 30 min each were necessary (3 sessions per week, on non-consecutive days). The cognitive development (through the WPPSI-IV), language (PLON-R), autistic traits (IDEA), and EF level (ENFEN) of the participant were assessed to determine their level in these aspects and thus adjust the type of responses and tasks (verbal or non-verbal) that would shape the intervention program to be designed. The results of these four tests determined the content of the intervention program to be designed.
- Once the initial evaluation was completed (right on the second day after its completion), a pre-intervention session of about 30 min duration was conducted. Tasks based on the DCCS task, Color–object Stroop task, and Mr. Cucumber task were administered. This session was recorded for subsequent analysis.
- Exactly one month after the pre-intervention session, the program implementation began; that is, the intervention started. A total of 18 intervention sessions were carried out (three per week), extending the intervention over 6 weeks. In each session, two tasks (verbal) were administered: one aimed at optimizing flexibility and another directed at inhibition, with their presentation order randomized. Each task lasted 8–13 min, with three minutes of rest between them. Thus, each intervention session lasted around 30 min. Each intervention session was entirely recorded.
- After completing the intervention (exactly on the second day after its completion), the post-intervention session was conducted. Three tasks similar to those administered in the pre-intervention session were administered (similar in the sense that they were based on the same tasks—DCCS task, Color–object Stroop task, and Mr. Cucumber task—but contained different stimuli; that is, animals, food, clothing, and everyday objects of the child presented as stimuli varied). The post-intervention session also lasted 30 min and was recorded.
- Three weeks after the post-intervention session, the maintenance session took place. It was similar to the pre- and post-intervention sessions in that, once again, three tasks based on the DCCS task, Color–object Stroop task, and Mr. Cucumber task were administered but comprised different stimuli from those used in the pre- and post-intervention sessions. The duration of this session was also 30 min and was also recorded. It is worth mentioning that although working memory was not affected (according to results obtained in the initial session), in the pre- and post-intervention sessions, as well as in the maintenance session, it was decided to evaluate working memory to check if, despite no intervention on it, it remained stable or showed any changes. This would also help to understand if, in case changes were detected between pre-, post-, and maintenance measures of cognitive flexibility and inhibition, it could be due to the effectiveness of the intervention or to the child’s own developmental process.

Figure 1 represents the procedure, intervention, and assessment tools used in each phase.

All sessions took place in the participant’s school context (although outside their regular classroom) and during regular school hours (specifically, during morning recess time). As mentioned earlier, all these sessions, except for the initial evaluations, were recorded and subsequently coded using the ad hoc constructed observation instrument and the LINCE PLUS recording tool. Coding was performed by two expert observers in observational methodology, education, child development, EF, and ASD. The observation conducted adhered to scientific criteria. It was active (as the aim was predetermined), non-participatory (the observers did not interact with the children), and direct (allowing complete perceptibility of behaviors from recorded footage) [28]. The data entered included information about the frequency and order of behaviors. The data were concurrent (behaviors could co-occur and belong to several dimensions of the observation instrument) and event-based (the behaviors were coded as they occurred, and thereby, the primary parameter used in the record was the order of events). Consequently, the data were Type II [76].

To ensure the quality of observational data, initially, a consensual agreement was carried out between the two authors of this manuscript. Subsequently, the first author coded all the observation sessions and randomly selected 10% of the entire corpus of videos to code them a second time and calculate intra-observer reliability. The second author randomly selected another 20% of the entire corpus of videos, different from those selected by the first author, and coded them to calculate inter-observer reliability. Intra- and inter-observer reliabilities were calculated using the generalizability theory [77] and the SAGT software. Specifically, for the calculation of intra-observer reliability, a design of three facets (Dimension, Categories, and Time) was used, considering Dimension and Categories as differentiation facets and Time as an instrumentation or generalization facet (Dimension, Categories/Time = DC/T). To calculate inter-observer reliability, another three-faceted design was used: DC/O (Dimension, Categories/Observer). In this case, Dimension and Categories were considered differentiation facets again, but now the instrumentation or generalization facet was the Observer. Regarding intra-observer reliability, in all sessions, the values of intraclass correlation coefficients (ICCs) were >0.95. Regarding inter-observer reliability, all ICCs were >0.91. Therefore, the quality of observational data was always very good [78,79].

Guidelines for Reporting Evaluations based on Observational Methodology (GREOM) [68] and the Methodological Quality Checklist for Studies based on Observational Methodology (MQCOM) [80] were followed.

### 2.4. Data Analysis

With the observational data referring to EF (working memory, flexibility, and inhibition) at each assessment moment (pre-, intervention, post, and maintenance), a lag sequential analysis was conducted [66]. Sequential analysis is one of the quantitative techniques most appropriate for analyzing qualitative data collected by direct observation [81]. Lag sequential analysis is a statistical procedure that allows finding significant behavior sequences, that is, chains of events that repeatedly occur in sequence data with a probability greater than mere chance (patterns of behavior). Thus, it determines whether and how events unfolding in time are related to each other [65,76,82,83,84]. Therefore, lag sequential analysis allows us to answer the following question: what tends to follow what? Or, in other words, it allows us to answer the question: given category A at time t, what category is more likely to appear at time t + 1? And at t + 2? And at t + 3? And at t + k? So, lag sequential analysis is very useful when our aim is to understand what happened during an intervention program, as it allows for the detection of regularities (patterns of behavior) at different points in time and the ability to compare them.

This procedure involves comparing the conditional probability of a particular “target” behavior at specific times before or after another “given” or “criterion” behavior with the average unconditional probability of that target behavior occurring. So, lag sequential analysis can be calculated in retrospective (moments prior to the “given” behavior) and prospective (moments after the “given” behavior) modes.

Thus, lag sequential analysis requires determination according to the needs of each study. This includes (1) the given or criterion behavior(s): those category(ies) of the observation instrument that are desired to know if they generate patterns of behavior; (2) the target or conditional behaviors: those categories of the observation instrument that are desired to know if they are associated with a probability greater than mere chance to the given or criterion behavior, thus forming the behavior patterns; (3) lags: distances or order within the conditioned behavior concerning the presence of the given behavior, either retrospectively, prospectively, or both.

The statistical parameter that allows for performing the calculations by comparing the conditional and unconditional probabilities of behaviors and therefore determining whether the relationship between the given behavior and each conditional behavior is significant at each lag is the adjusted residuals [85].

After obtaining the adjusted residuals, the behavioral pattern(s) have to be “constructed”, beginning with the specified criterion behavior in each instance. In each lag (whether positive or negative), the associated behaviors with a significant adjusted residual value will be incorporated: >1.96 for activation relationships and <−1.96 for inhibitory relationships (at a significance level of *p* < 0.05).

Researchers can consider where the structure (the pattern) conventionally ends, i.e., to end the interpretation purposes of the obtained structure. The following interpretation guidelines should be applied: (a) when there are no more lags with statistically significant behaviors, (b) when there are two consecutive empty lags, or (c) when there are several statistically significant behaviors in two consecutive lags, and the first of the lags is considered the MAX LAG [66,75,81]. Diachronic analysis of qualitative data. Therefore, the significant behaviors that appear in this first lag would constitute part of the pattern or patterns but not those that appear in the next lag.

Lag sequential analysis has been shown to be useful for exploratory studies where specific a priori hypotheses about expected patterns are not specified [83,86]. This is the case in our study. Substantially, lag sequential analysis has shown to be useful in studies conducted on child development, more specifically on child cognitive development [29] and on development in children with ASD [53,70,87].

According to the objective of this study, a prospective sequential analysis (lags +1 to +5) was carried out adopting all categories of the observation instrument except for the Actor criterion behaviors (since their categories—child or adult—always co-occurred with some of the other categories of the observation instrument, so they would not provide extra information in possible patterns). The GSEQ 5.1 program was used for sequential analysis.

For the data analysis in this study, the 18 intervention sessions were grouped into three blocks: 1st intervention moment (sessions 1 to 6); 2nd moment (sessions 7–12); and 3rd moment (sessions 13–18). In each moment, two tasks aimed at optimizing flexibility and two aimed at inhibition were randomly selected. Within each of these three intervention moments, the recording of each type of task (the two referring to flexibility on one hand and the two referring to inhibition on the other) were combined to extract the behavioral patterns that characterized child performance regarding each EF targeted in each of these three moments.

In total, considering the different EF and assessment moments, 15 sequential analyses were performed corresponding to the following sessions: pre-, post-, and maintenance referring to working memory; pre-intervention and interventions 1, 2, and 3; post-intervention and maintenance for flexibility and inhibition.

Subsequently, the patterns referring to each EF obtained at different assessment moments were compared, thus being able to understand the development and change, or lack thereof, in child performance and therefore, obtaining information about the program’s effectiveness.

## 3. Results

Figure 2, Figure 3, Figure 4, Figure 5, Figure 6, Figure 7, Figure 8, Figure 9, Figure 10, Figure 11, Figure 12, Figure 13, Figure 14, Figure 15 and Figure 16 show the patterns obtained in different moments of the intervention in the three executive functions evaluated: Working memory, flexibility and inhibition.

In these figures, letters CrB are equivalent to Criterion Behavior; L is equivalent to Lag; Blue line is an empty lag and a red arrow means sequential behavior. 

### 3.1. Behavioral Patterns in Working Memory Tasks: Pre-Intervention, Post-Intervention, and Maintenance Session

**Figure 2:**
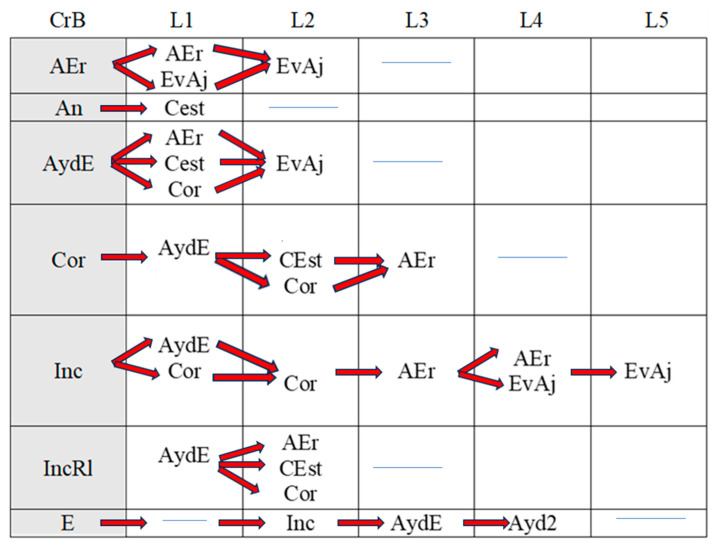
Working memory task patterns: pre-intervention session.

In the pre-intervention session (Figure 2), the boy correctly executed successive series of working memory tasks (Cor), although he also failed to complete some of the tasks adequately (Inc). Despite these errors, however, and with the help of the adult present during the sessions (AydE), the boy changed his strategy (CEst), corrected his errors (AEr), and performed an adjusted evaluation of the execution of the task (EvAj).

**Figure 3:**
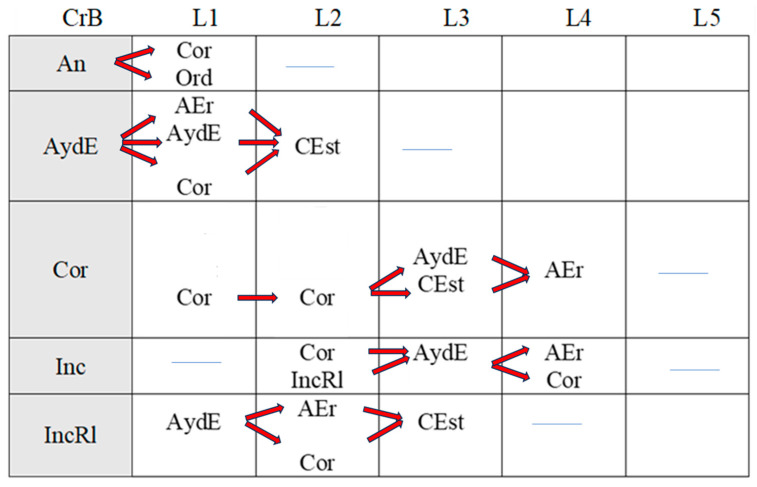
Working memory task patterns: post-intervention session.

In the post-intervention session (Figure 3), the boy continued to correctly resolve tasks (Cor), and we observed a slight improvement in his execution of incorrect tasks (Inc) in that he came closer to the solution than before (IncRl). Again, with the help of the adult (AydE), the boy changed his strategies and corrected his errors (AEr).

**Figure 4:**
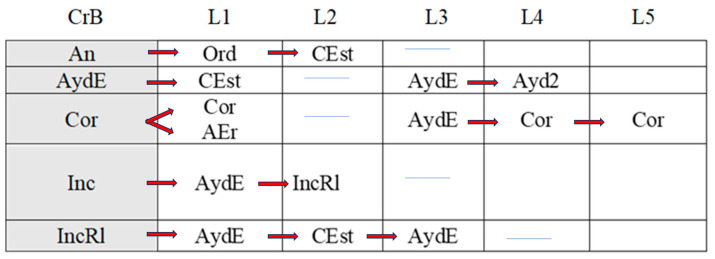
Working memory task patterns: maintenance session.

In the maintenance session (Figure 4), the boy correctly executed working memory tasks (Cor) with the help of the adult (AydE). Incorrect executions with adult help were closer to correct executions (IncRl).

In brief, the boy performed similarly during the pre-intervention, post-intervention, and maintenance sessions in working memory tasks. His actions (change of strategies, self-correction, and correct executions) can be considered satisfactory, although adult intervention was sometimes necessary to bring about improvement.

### 3.2. Behavioral Patterns in Flexibility Tasks: Pre-Intervention, Early-, Mid-, and Late-Stage Intervention, Post-Intervention, and Maintenance

**Figure 5:**
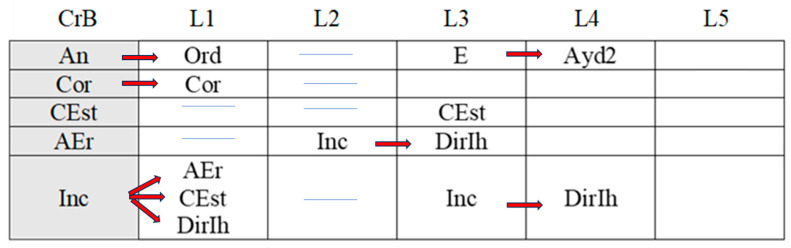
Flexibility task patterns: pre-intervention session.

In the pre-intervention session (Figure 5), the child began to resolve the tasks before being told to by the adult (An), and the child waited (E) because the adult instructed him to wait (Or). He performed some of the tasks correctly (Cor), tried new strategies (CEst), and corrected errors (AEr), but with limited success, as he subsequently failed to execute the same tasks correctly.

**Figure 6:**
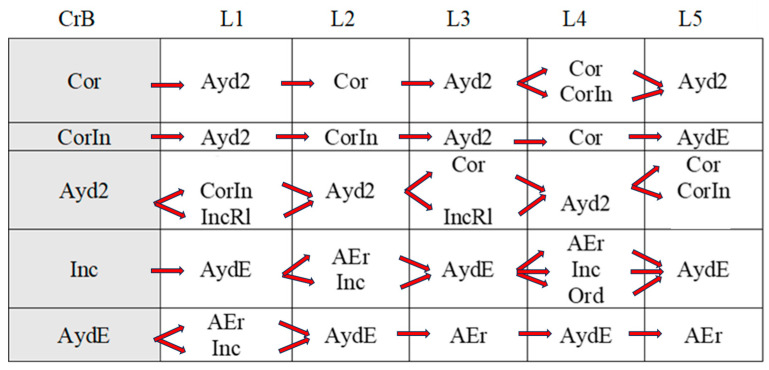
Flexibility task patterns: early-stage intervention.

In the early-stage intervention sessions (Figure 6), the boy waited to be told to start the tasks, and there were no anticipatory behaviors (An), indicating an improvement in self-control. He correctly performed some of the tasks (Cor) but with indirect help from the adult (Ayd2). We also observed successive series of correct yet incomplete executions (CorIn) that he repeated, despite indirect help from the adult (Ayd2). This help (Ayd2) only resulted in correct executions (Cor) on counted occasions.

Incorrect executions (Inc) were followed by direct help from the adult (AydE). This help (AydE) was effective in some cases, resulting in self-correction (AEr), but in other cases, it was not, as the boy continued to perform the task incorrectly (Inc).

Changes in strategy (CEst) and self-corrections (AEr) did not generate any patterns, indicating that they were isolated in time and not accompanied by any of the other actions analyzed.

**Figure 7:**
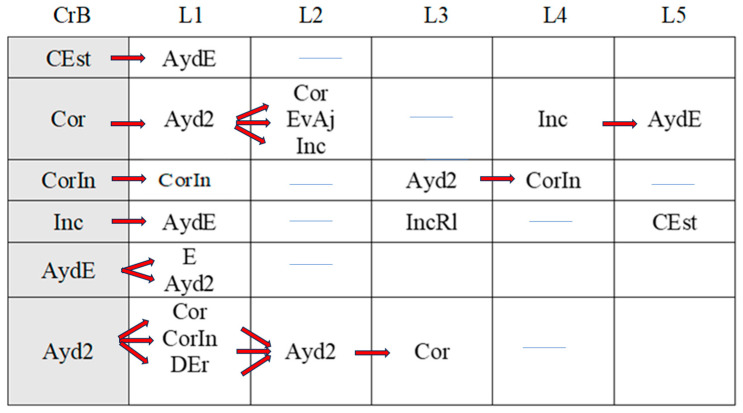
Flexibility task patterns: mid-stage intervention.

In the mid-stage intervention sessions (Figure 7), the child continued to exert self-control and did not start the tasks ahead of time (An).

There were isolated changes in strategy (CEst) followed by direct guidance (AydE) from the adult, which did not appear to be effective, as it was not followed by any specific actions by the boy.

Indirect help from the adult (Ayd2) resulted in successful task executions (Cor), which were followed by correct executions (Cor) and, for the first time, metacognitive and adjusted evaluations by the boy (EvAj). There were, however, also some incorrect executions (Inc), which were followed by direct help from the adult (AydE).

The boy also performed correct yet incomplete executions (CorIn) that, despite indirect help from the adult (Ayd2), were not always followed by successful completion of the task (CorIn).

There was, however, some improvement in incorrect executions (Inc) in that direct intervention from the adult (AydE) helped the boy to change strategies (CEst) and come closer to the solution, even though he did not completely resolve the task (IncRl).

Indirect help from the adult (Ayd2) was common during these mid-intervention sessions and led to improvements in the boy’s actions, with the appearance, for the first time, of self-detection of errors (DEr). Of note was the absence of patterns related to these self-corrections (AEr).

**Figure 8:**
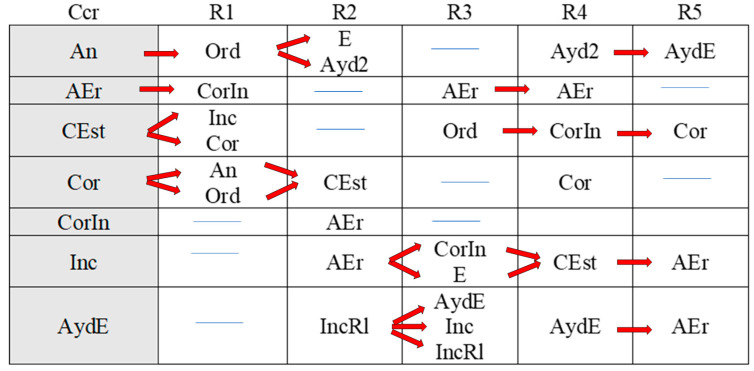
Flexibility task patterns: late-stage intervention.

In the late-stage intervention sessions (Figure 8), although we detected some deterioration in the progress that had been made in the boy’s actions (An)—the adult needed to re-intervene to stop the boy (Ord) starting the task before he was instructed to (E)—considerable progress was observed.

The child also employed unsuccessful changes of strategies (CEst), but with the help of the adult (Ord), they led to correct executions (CorIn, Cor).

The boy continued to complete the tasks successfully (Cor), and there was an increase in cognitive complexity as incorrect executions (Inc) and correct yet incomplete executions (IncRl) were followed by self-correction (AEr). In other words, the boy came closer to the correct answer and continued to demonstrate flexibility in terms of trying out new strategies to find the solution (CEst). Direct help from the adult (AydE) also favored self-correction (AEr) and led to improved actions.

**Figure 9:**
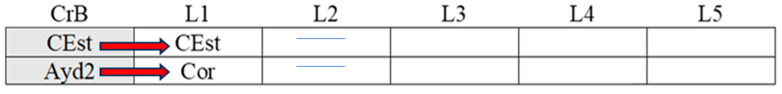
Flexibility task patterns: post-intervention session.

In the post-intervention session (Figure 9), the boy’s actions stood out for their quality. He exerted self-control—the absence of patterns related to premature starts(An)—and changed strategies without the intervention of the adult (CEst), suggesting improvements in cognitive flexibility.

Of note was an absence of patterns generated by self-corrections (AEr) and executions, regardless of whether they were correct (Cor), correct but incomplete (CorIn), or incorrect (Inc).

Indirect help from the adult (Ayd2) led to correct executions (Cor).

**Figure 10:**
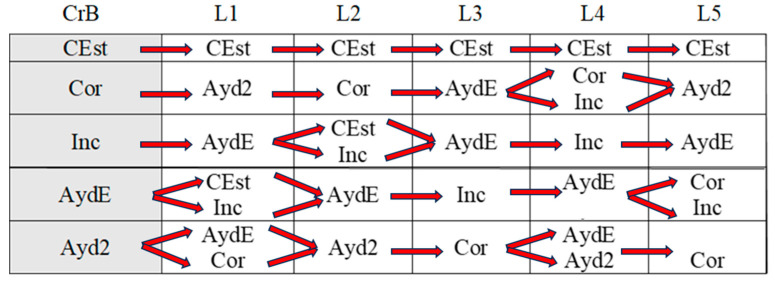
Flexibility task patterns: maintenance session.

The boy continued to exert self-control—the absence of anticipation (An)—in the maintenance session (Figure 10), and there was a continued absence of self-correction (AEr).

Like the post-intervention session, the boy continued to try out new strategies throughout the task (CEst) without guidance from the adult. The increased frequency of strategy changes possibly reflects progress in terms of cognitive flexibility.

Correct executions (Cor) occurred alongside incorrect executions (Inc) despite help from the adult (AydE), indicating the need for a longer intervention program to consolidate the achievements made.

Just as in the post-intervention stage, correct yet incomplete executions (CorIn) did not generate patterns. Incorrect executions, however, seemed to indicate a certain deterioration. Despite the adult’s help (AydE), the boy was not only unable to find the correct answer (Cor) but also completed the task incorrectly on repeated occasions (Inc), just as in the pre-intervention and early intervention sessions. This again would suggest that more intervention sessions are needed to build on the improvements observed at the end of the intervention.

Direct help from the adult (AydE) was still necessary in the maintenance stage. The adult guided the boy towards finding another strategy to resolve the problem (CEst), but despite the slight progress made, he still needed the adult’s help to correct the error (AydE) and complete the task correctly (Cor), although he did not always manage to do so (Inc). Future research should analyze which type of adult help is most effective in terms of leading to the correct execution of tasks by children.

Indirect help from the adult (Ayd2) continued to be necessary to achieve correct executions in the maintenance session, but the number of correct executions (Cor) was higher than in previous sessions, indicating that the intervention program was a success. The improvements detected in other aspects, however, did not appear to have been maintained, again suggesting the need for longer interventions.

### 3.3. Behavioral Patterns in Inhibition Tasks: Pre-Intervention, Early-, Mid-, and Late-Stage Intervention, Post-Intervention, and Maintenance

**Figure 11:**
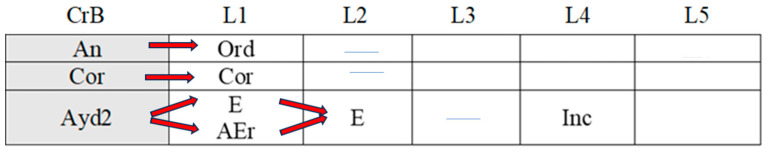
Inhibition task patterns: pre-intervention session.

The boy started the inhibition tasks ahead of time (before the instruction from the adult) in the pre-intervention session (Figure 11), generating a pattern that indicated a lack of self-control (An). We also detected a successive, but not continuous, pattern of correct executions (Cor). Neither incorrect (Inc) nor correct yet incomplete (CorIn) executions generated patterns, indicating successful task resolution.

Indirect guidance from the accompanying adult (Ayd2) helped the boy wait (E) until instructed to begin the task, regardless of whether this was completed unsuccessfully (Inc). It also helped the boy correct his errors (AEr), even though he subsequently failed to complete the task successfully (Inc).

**Figure 12:**
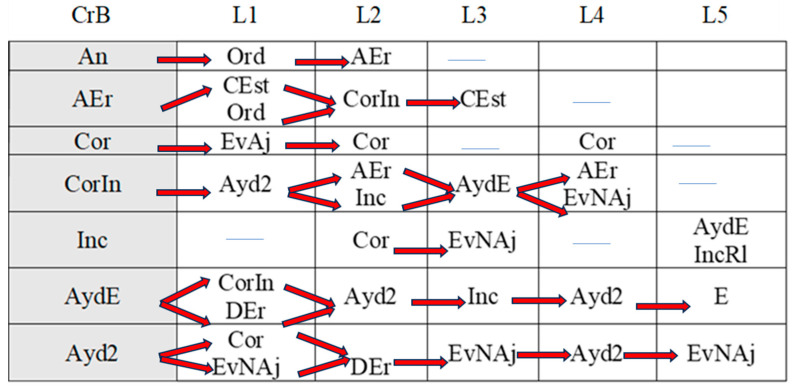
Inhibition task patterns: early-stage intervention.

In the early intervention stage (Figure 12), the premature initiation of tasks (An) was followed by instructions from the adult (Ord) to help the child correct his errors.

The boy corrected his errors (AEr), changed strategies (CEst), and came close to correcting yet incomplete executions (CorIn), although he sometimes needed previous instructions from the adult (Ord). He continued to try out new strategies (CEst) when he did not succeed in fully completing the task (CorIn).

Correct executions (Cor) were accompanied by adjusted evaluation (EvAj), indicating adequate action.

Correct yet incomplete executions (CorIn) were followed by indirect help from the adult (Ayd2), which appeared to help the boy correct his errors (AEr) in some cases, but in others not, as it was followed by incorrect executions (Inc). These actions were accompanied by direct help from the adult (AydE), which, again, contributed to satisfactory executions (self-correction) on occasions (AEr). On other occasions, however, this assistance did not have this effect, as it was followed by non-adjusted evaluations (EvNAj).

Direct help from the adult (AydE) appeared to be effective, as it helped the boy to perform a task correctly albeit incompletely (CorIn) or to recognize/detect a mistake (AEr). Nonetheless, he required the indirect help of the adult (Ayd2) to continue the activity, but in this case, it was not effective, as it was followed by an incorrect execution (Inc).

Indirect help from the adult (Ayd2) was sometimes immediately followed by adequate task resolution actions—correct executions (Cor) and detection of errors DEr)—but these were then followed by non-adjusted evaluations (EvNAj) despite the repeated assistance from the adult (Ayd2). On other occasions, these non-adjusted evaluations (EvNAj) were present from the beginning and were continuously alternated with indirect help from the adult (Ayd2).

**Figure 13:**
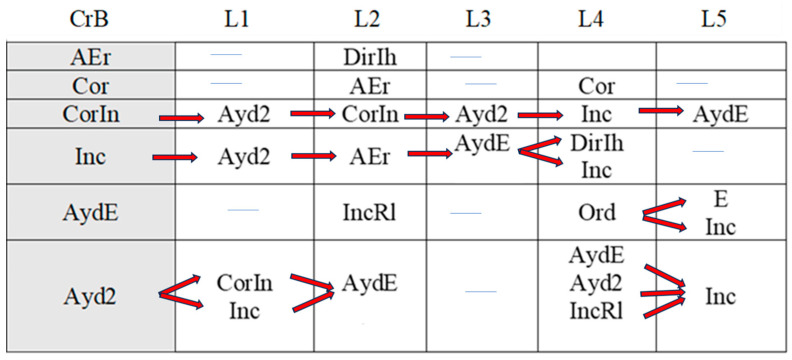
Inhibition task patterns: mid-stage intervention.

In the mid-stage intervention sessions (Figure 13), the boy exhibited good self-control, as he did not start the activity ahead of time (An). He also corrected his errors (AEr) and inhibited responses (DirIh). Correct executions (Cor) were followed by self-corrections (AEr), which resulted in a higher number of correct executions (Cor).

Incorrect executions (Inc) were followed by help from the adult (Ayd2), which helped the boy correct his errors (AEr) and, on occasion, even adequately inhibit responses (DirIh). On other occasions, however, despite the help, the child failed to perform the task correctly (Inc).

Both direct (AydE) and indirect help (Ayd2) from the adult generated patterns of actions that resulted in incorrect executions (Inc, IncRl, and CorIn), despite frequent and varied help from the adult.

**Figure 14:**
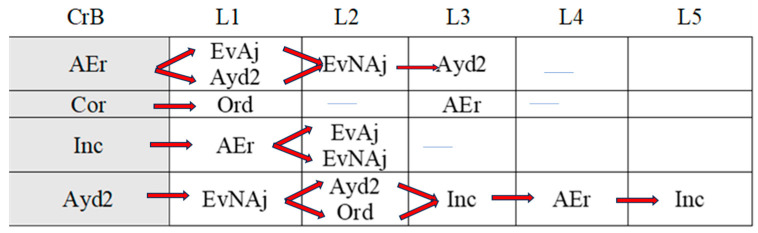
Inhibition task patterns: late-stage intervention.

During the late-intervention stage (Figure 14), the boy continued to exhibit self-control, i.e., he did not begin the tasks until instructed to by the adult (An). Nevertheless, self-corrections (AEr) were followed by both adjusted (EvAj) and non-adjusted evaluations (EvNAj) and by help from the adult (Ayd2).

Interestingly, correct executions (Cor) stopped occurring successively, and unlike at other stages of the intervention, they were followed by instructions from the adult (Ord) that led to self-correction (AEr).

Incorrect executions (Inc) were followed by self-corrections (AEr) as well as both adjusted (EvAj) and non-adjusted evaluations (EvNAj).

Direct help from the adult (AydE) did not generate a pattern, unlike indirect help (Ayd2), although this assistance did not appear to be overly effective. Although the child corrected his errors (AEr), these were followed by non-adjusted evaluations (EvNAj) and incorrect executions (Inc), similar to those (Inc) that had preceded the error correction (AEr).

**Figure 15:**
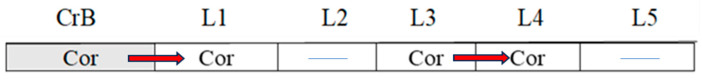
Inhibition task patterns: post-intervention session.

In the post-intervention session (Figure 15), the boy continued to exhibit self-control, and we therefore did not detect any patterns related to the premature initiation of tasks (An). He also continued to correctly execute tasks in succession (Cor), possibly explaining why correct yet incomplete executions (CorIn), incorrect executions (Inc), or adult help (AydE and Ayd2) did not generate patterns. The boy therefore performed adequately and autonomously to resolve inhibition tasks post-intervention.

**Figure 16:**
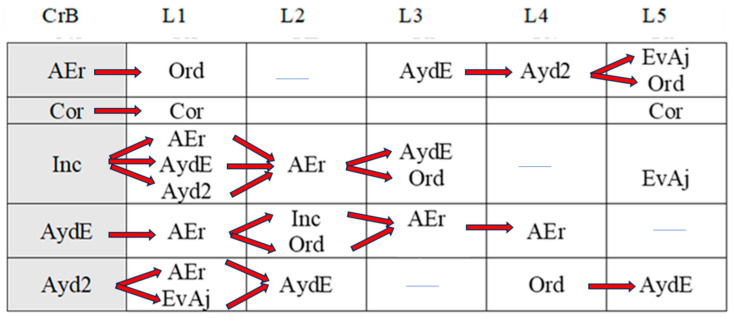
Inhibition task patterns: maintenance session.

In the maintenance session (Figure 16), self-control behaviors, the absence of premature task initiation (An), and correct executions (Cor) were observed, and correct yet incomplete executions (CorIn) did not generate patterns.

By contrast, self-correction (AEr) and incorrect executions (Inc) now generated patterns, unlike in the post-intervention session. The same situation was observed with adult help (AydE and Ayd2). The above patterns indicate the need for a different form of adult help to channel the child’s actions towards self-correction of errors (AEr) and adjusted evaluations (EvAj).

In brief, we observed that the improvements detected after the intervention, which were particularly evident in the form of adequate levels of self-control and correct task execution without help, were not completely maintained, as adult intervention was needed again in the maintenance phase to guide the boy’s actions.

## 4. Discussion and Conclusions

The hypothesis postulated was that cognitive intervention would positively impact the child’s EFs (working memory, flexibility, and inhibition). They will be improved in terms of complexity and elaboration. The results obtained partially corroborated this hypothesis because the executive functions of flexibility and inhibition had improved in the post-intervention evaluation, but this improvement was barely maintained after three weeks without intervention. However, working memory (which the intervention did not focus on) remained stable from the beginning to the end of the intervention. This indicates that the intervention was effective in improving deficient executive skills in the child (flexibility and inhibition) and that the mixed-methods approach chosen was highly suited to evaluating the intervention. These results are consistent with reports in the literature that executive functions are modifiable and can be improved throughout an individual’s life, even in childhood [1], and specifically in children with ASD [25]. The results are also in line with reports that show the benefits of using a mixed-methods approach to analyze complex processes like human development in greater depth and from complementary stances [88].

Our findings suggest, however, that to achieve better and more stable results, (1) the duration of the intervention should be extended—despite the fact that our intervention is longer in time than others in the literature that were shorter (4 weeks, for example) and obtained similar results [89] and (2) the complexity of the tasks gradually increased, as the repeated and continuous use of executive functions beyond a level that is considered comfortable is key to improvement [1]. Patterns in the maintenance session showed that effects were barely maintained after three weeks without intervention. These results coincide with other studies suggesting that the effects fade once the intervention ends, therefore highlighting the need to continue training executive functions [3,89]. The decrease in executive functions after a period of non-intervention in children with ASD has been evidenced in the literature regardless of the intervention method used (cognitive intervention, virtual training, mindfulness, or movement-based training), the number and duration of the sessions, the age of the children, or their severity level [25,89,90]. Why this decrease happens is still unclear. Numerous studies postulate that EF difficulties in individuals with autism stem from aberrations in the brain’s system. Children with autism exhibit reduced connectivity among diverse brain areas, impairing complex information processing [25,91]. The principles of experience-dependent neuroplasticity explain that the brain continuously remodels its neural circuitry in response to one’s experience in changing environments. A particular external change in the environment playing an important role in learning development is the massed practice (intervention), resulting in neural change via the strengthening of functional neural pathways that results in both broad and specific gains in related behaviors [92]. Furthermore, features of this mass practice involving intervention that are considered essential to maximizing outcomes include salience, hierarchically graded tasks, adaptive difficulty levels, and repetition [93]. Results from the literature also show the benefits of one-to-one intervention [94] and interaction with adults [25,93]. In addition, interventions are characterized by constituting a positive and reinforcing context that can facilitate the learning of the desired executive functions [90]. All these elements were included in our designed intervention. This massed practice and these positive elements disappear after stopping the intervention and, due to the lack of stimulation, the strengthening of neuronal pathways decreases and, consequently, executive functions decline. Nevertheless, further research is needed to provide additional explanation and evidence concerning these mechanisms [90,92].

The relevance of one-to-one intervention [94] and interaction with the adult [25]—both key elements included in our designed intervention, as previously mentioned—highlights the need for further analysis of the intervention strategies employed by the adult at the different stages of the program. This analysis is required to investigate which strategies are most effective for improving activity and to determine the extent to which the results can be extrapolated to other home and school settings.

A limitation of the study is the size sample, which limits the generalizability of the findings. As mentioned, this is a pilot, single case carried out with one child. However, while limiting the generalizability of the results, this also has benefits. One of the benefits of conducting a pilot study is that it allows for the improvement or replacement of components and activities within the intervention [95,96]. In our research, also taking into account that the designed intervention is aimed at minors (and, furthermore, minors with developmental difficulties), testing the designed intervention with a single participant before investing a large amount of time, financial, personal, and material resources to implement it with more participants is fair and responsible. This is consistent with international ethical principles and allows for the optimization of personal, temporal, and economic resources. The value and relevance of this type of study are evident in the literature, highlighting not only the benefits of this type of study but also encouraging more discussion and dissemination of pilot studies. Researchers are therefore urged to report on their pilot study [95,96,97,98,99]. In the same sense, other authors [100,101] defend pilot studies and the need to carry out, disseminate, and value them by considering them an integral part of the research procedure. This study also constitutes a response to this demand and appeal, showing the value of pilot studies in education research through practice, and further taking into account that pilot studies focused on educational research is a field not yet fully explored [96]. Given that we are dealing with a single-case study, the circumstances are quite analogous. Although this type of study acquired negative connotations in the literature some time ago and obviously implies an inherent weakness regarding the lack of representativeness and non-replication of results (given its own aim), nowadays, single-case studies are recognized as a valuable opportunity for conducting rigorous and intensive studies into human behavior [33]. The logic of a single case is intra-case by nature [102]. It focuses on the results of one single case, which is studied in depth and allows us to consider the diachronic perspective while also highlighting the complexity and richness of the real-world context in which the phenomenon occurs [33]. Other single cases carried out with children with autism can be found in the literature [33,34], showing the usefulness of this type of study to assess the effectiveness of different types of interventions aimed at a child with autism. Coherently with the arguments and explanations that are being presented, an observational methodology was used in this study. It is important to highlight that an observational methodology is intensive (not extensive), or in other words, it is focused on an idiographic assessment and not on a nomothetic one. This approach necessitates working with one observational unit or limited observational units while enabling the collection of a substantial volume of highly accurate data. In studies conducted within an observational framework—such as the present one—the focus is primarily on providing a comprehensive account of the natural behaviors exhibited by one observational unit or limited observational units rather than on the representativeness of these behaviors within more observational units [28,59,103]. Consequently, it is crucial that observational studies are not unjustly evaluated through the lens of experimental methodologies, which remains a dominant paradigm in scientific research. This “unjust and misguided evaluation” has led to the occasional dismissal of this approach in the study of child development and learning despite its status as the optimal methodology [30,104].

We consider that another important contribution of this study can also be found in the creation of an ad hoc observation instrument—as it was explained in the Instrument Section, it is a characteristic and requirement of systematic observation [28,59]. This study offers a free observational tool that allows observational data on the functioning of children to be captured in a natural and objective way. This assessment instrument is suitable for collecting quality data (the reliability results in the data quality control analysis support the adequacy of the data obtained) on the functioning of children during interventions without altering the dynamics of their interactions. In this line, the literature underlines the importance of developing low-cost instruments specifically designed to observe the child’s behavior in a real-life context to propose personalized interventions based on the differential functioning of children [105]. In this sense, it is noteworthy that this ad hoc observation instrument can also be useful for other researchers and professionals, who can adapt or adjust it to the characteristics of the context and individual to be evaluated in each case. Although it is true that this observation instrument can never be used as is in its entirety in other studies (due to the variety of spontaneous behaviors that a person can perform and the different characteristics that a natural context can present), it can serve as a starting point—it can be adapted or adjusted in all or some of its criteria and/or categories—for other studies with similar objectives. In this sense, we consider that it can be especially useful for educators, who frequently affirm they do not have the time or training necessary to build an adequate observation instrument that allows the objective and precise collection of the relevant behaviors necessary for an adequate evaluation of their students. Educational and ASD professionals need new methods and instruments to evaluate the interventions and the changes in the children [106]. Our observation instrument contributes to responding to this need.

This study also shows that this observation instrument could be useful for sequential analysis. Similarly to other authors [106], we established that the use of observational methodology offers the opportunity to obtain a large amount of data that can be analyzed with different techniques—such as sequential analysis—in a more sensitive and detailed manner, offering information to which questionnaires frequently used are not sensitive [106].

Another highlight of this study is the longitudinal perspective adopted. Although it is highly practical and adequate for examining the short-, medium- and long-term effectiveness of intervention processes, literature is not consistently employed due to the costs embroiled [107,108]. Nevertheless, it provides valuable insights that are particularly pertinent and relevant to the domain of intervention in childhood, especially considering the changes that are intended to be achieved in this critical period of life that can condition subsequent development. In the context of this research, the acquisition of such information has been facilitated through the implementation of systematic observation, which, as mentioned, is a complex yet optimal method for conducting an in-depth analysis of child behavior [29,30,31,32,33,34,35,36,37,38,39,40,41,103]. This work adds to the research that highlights the importance of early intervention to develop functional skills in children with ASD [90,109]. The designed intervention can serve as an example for other professionals, adjusting it to the characteristics of each child since it is already known that each intervention must be individualized according to the needs and characteristics of each child [90,105,107]. In this sense, this intervention can serve as help and example to encourage teachers and other educational professionals to apply research-based practices to improve EF in children with ASD. Furthermore, in the same line, the observation instrument that was developed, as indicated, can serve as a valuable resource for teachers and other educational professionals to objectively, precisely, and thoroughly assess whether an intervention is achieving its intended purpose.

In brief, this pilot study is an example of how assessment and intervention in ASD through a mixed-methods approach assessing the entire intervention process, including maintenance measures of the effects of the intervention, can achieve significant and real changes in the quality of life of children with ASD. This issue is directly related to the Sustainable Development Goals 3—healthy lives and well-being—and 4—quality education—which were proposed by all United Nations Member States in 2015 as part of the 2030 Agenda for Sustainable Development [110].

## Figures and Tables

**Table 1:** Observation instrument.

Criterion	Category	Code
Actor	Adult	A
	Boy	N
Previous actions by boy	Starts ahead of time	An
	Waits	E
Task tackling strategies employed by boy	Changes strategy	CEst
	Perseveres	Per
Executions by boy	Correct	Cor
	Correct yet incomplete	CorIn
	Incorrect	Inc
	Incorrect yet related	IncRI
Type of response by boy	Direct inhibited	DirIn
Metacognitive regulation by child: monitoring	Self-correction of error	AEr
	Detection of error	DEr
	Non-detection of error	NDEr
Metacognitive regulation by child: evaluation	Adjusted evaluation	EvAj
	Non-adjusted evaluation	EvNAj
Previous actions by adult	Orders execution of an action	Ord
Intervention by adult	Indirect help gives clues	Ayd2
	Direct help corrects execution	AydE

## Data Availability

The data presented in this study are available upon reasonable request from the corresponding author. The data are not publicly available due to privacy restrictions.
